# Frequent convergence of *mcr-9* and carbapenemase genes in *Enterobacter cloacae* complex driven by epidemic plasmids and host incompatibility

**DOI:** 10.1080/22221751.2022.2103456

**Published:** 2022-08-05

**Authors:** Tingting Xu, Chun-Xu Xue, Yuxin Chen, Junxi Huang, Weiyuan Wu, Yuemei Lu, Qiuhui Huang, Dandan Chen, Kai Zhou

**Affiliations:** a Shenzhen Institute of Respiratory Diseases, Second Clinical Medical College (Shenzhen People's Hospital), Jinan University; the First Afﬁliated Hospital (Shenzhen People’s Hospital), Southern University of Science and Technology, Shenzhen, People’s Republic of China; b Department of Laboratory Medicine, Nanjing Drum Tower Hospital Clinical College of Jiangsu University, Nanjing, People’s Republic of China; c Clinical Laboratory, Second Clinical Medical College (Shenzhen People's Hospital), Jinan University; the First Afﬁliated Hospital (Shenzhen People’s Hospital), Southern University of Science and Technology, Shenzhen, People’s Republic of China

**Keywords:** *Enterobacter cloacae* complex, *mcr-9*, carbapenemase, epidemic plasmid, antibiotic resistance

## Abstract

Convergence of *mcr* and carbapenemase genes has been sporadically detected in *Enterobacter cloacae* complex (ECC) with an upward trend. However, the state of the epidemic and underlying mechanism of such convergence has been poorly understood. In this study, the co-occurrence of MCR and carbapenemases was systematically analyzed in 230 clinical ECC isolates collected between 2000 and 2018 together with a global dataset consisting of 3,559 ECC genomes compiled from GenBank. We identified 48 *mcr-9*/*mcr-10*-positive isolates (MCR-ECC) (20.9%) in our collection, and a comparable ratio of MCR-ECC (720/3559, 20.2%) was detected in the global dataset. A high prevalence of carbapenemase-producing MCR-ECC (MCR-CREC) was further identified in the MCR-ECC of both datasets (16/48, 33.3%; 388/720, 53.9%), demonstrating a frequent convergence of *mcr-9/10* and carbapenemase genes in ECC worldwide. An epidemic IncHI2/2A plasmid with a highly conserved backbone was identified and largely contributed to the dissemination of *mcr-9* in ECC worldwide. A highly conserved IncX3-type NDM-1-carrying plasmid and IncN-type IMP-4-carrying plasmid were additionally detected in MCR-CREC isolated in China. Our surveillance data showed that MCR-CREC emerged (in 2013) much later than MCR-ECC (in 2000), indicating that MCR-CREC could be derived from MCR-ECC by additional captures of carbapenemase-encoding plasmids. Tests of plasmid stability and incompatibility showed that the *mcr-9/mcr-10*-encoding plasmids with the NDM-1-encoding plasmids stably remained in ECC but incompatible in *Escherichia coli*, suggesting that the convergence was host-dependent. The findings extend our concern on the convergence of resistance to the last resort antibiotics and highlight the necessity of continued surveillance in the future.

## Introduction

Clinical infections caused by multidrug-resistant (MDR) bacteria with an increasing trend have become a critical threat to the public health network [1]. In particular, the dramatic increase of carbapenem resistance worldwide has greatly compromised the efficacy of carbapenems and prompted renewed attention to the importance of the last-line antibiotics colistin (polymyxin E). However, with the heavy consumption of colistin in veterinary medicine and clinical settings, colistin-resistant isolates have emerged globally [2–4], causing a therapeutic challenge in the clinical setting.

Colistin resistance was thought to be intrinsic in the past until the report of the first mobilized colistin resistance (*mcr*) gene *mcr-1* in 2015 [5]. Since then, an addition of nine *mcr* genes has been described and was assigned new numerical designations, namely *mcr-2* ∼ *mcr-10* [6–14]. Plasmid-mediated *mcr* genes have been widely spread in various bacterial species, the vast majority belonging to *Escherichia coli*, followed by other Enterobacteriaceae (e.g. *Klebsiella pneumoniae*, *Enterobacter* spp*.*, and *Salmonella enterica*), and *Aeromonas hydrophila* [9, 11, 15–17]. Currently, *mcr-1* is the most widely disseminated member of the *mcr* family, followed by *mcr-9*, *mcr-3*, and *mcr-5* [18]. Up to now, all *mcr* genes except for *mcr-6* are frequently harboured on conjugative plasmids or ColE-type plasmids mobilizable with helper plasmids [9, 19]. The *mcr-1*-harboring plasmids have been extensively studied and assigned to a large number of incompatibility groups, of which IncHI2, IncX4, and IncI2 were the predominant ones responsible for the dissemination of the *mcr-1* gene among *E. coli* isolates [20]. Most of the *mcr-9*-carrying plasmids were typed to be IncHI2 [17], while the type of *mcr-5*-encoding plasmids was highly diverse [8, 21].

*E. coli* and *K. pneumoniae* are the major reservoirs for most of the *mcr* genes, while *mcr-9* and *mcr-10* have been frequently reported in *Enterobacter cloacae* complex (ECC) [14, 16, 22, 23]. ECC is an important nosocomial pathogen capable of causing a wide variety of infections, such as pneumonia, urinary tract infections, and septicemia [24]. Molecular typing methods have identiﬁed more than 20 species/phylogenetic clusters in the complex [25]. Epidemiological studies show that *Enterobacter hormaechei* is the predominant species among ECC in the clinical setting, contributing to increased morbidity and mortality in hospitalized patients, particularly when infected by carbapenem-resistant isolates [24, 26].

Recently, the co-occurrence of carbapenemases and MCR in a single strain has been reported sporadically [27, 28], especially the combination of genes encoding metallo-beta-lactamases and MCR-9. Up to date, such genotype (i.e. coexistence of carbapenemase genes and *mcr-9*) has been found in *Enterobacter* spp., *Klebsiella* spp., *Escherichia* spp., and *Citrobacter* spp. [17, 22, 29–31]. An MDR *E. hormaechei* isolate co-harboring *bla*_NDM-1_ and *mcr-9* genes was identified in a retrospective screen of carbapenemase- and MCR-producing *Enterobacterales* recovered from a tertiary hospital in Hangzhou, China [30]. Two carbapenemase genes (*bla*_IMP-4_ and *bla*_NDM-1_) were simultaneously detected in an *mcr-9-*positive *E. hormaechei* strain [32]. In particular, a high incidence of carbapenemases, especially metallo-beta-lactamases, in *mcr-9*-positive genomes was detected in *Enterobacter* spp. collected from a tertiary hospital recently [33]. These data consistently suggest that *Enterobacter* spp. could be an important reservoir for carbapenemase genes and *mcr-9*. However, the underlying mechanism involved has been poorly understood. Herein, we aimed to systematically study the co-existence of *mcr* and carbapenemase genes in ECC isolates collected between 2000 and 2018 in a tertiary hospital of China together with a global dataset retrieved from GenBank. The plasmidom encoding *mcr* and carbapenemase genes were dissected, and their incompatibility and stability were tested to explore the co-occurrence mechanism.

## Materials and methods

### Bacterial isolates, mcr-positive-ECC (MCR-ECC) bacteria identification, and clinical data collection

ECC isolates were collected at a tertiary hospital of China between 2000 and 2018, and have been species-typed previously [34]. The presence of *mcr* genes including *mcr-1* ∼ *mcr-10* were assessed by using primers reported previously [16]. The *mcr* positive isolates were determined as MCR-ECC. Metadata including patients’ gender and age, dates of specimen collection, and specimen types were recorded.

### Antimicrobial susceptibility testing

The minimum inhibitory concentrations (MICs) of 14 antibiotics were evaluated according to the guidelines of the Clinical and Laboratory Standards Institute (CLSI) (M100-S30, 2020). MICs of colistin and tigecycline were determined by the broth dilution method, and the results were interpreted according to European Committee on Antimicrobial Susceptibility Testing (EUCAST) (version 10.0) criteria (https://eucast.org/clinical_breakpoints/). The other 12 antibiotics were determined using the agar dilution method. *E. coli* ATCC 25922 and *Pseudomonas aeruginosa* ATCC 27853 were used as quality control standards. MCR-ECC isolates resistant to imipenem and/or meropenem (MIC ≥ 4 mg/L) were determined as carbapenemase-producing MCR-ECC (MCR-CREC).

### Whole genome sequencing (WGS) and data analysis

Genomic DNA was extracted from all of MCR-ECC isolates using a Gentra Puregene Yeat/Bact. Kit (Qiagen, San Francisco/Bay area, CA, USA) and subjected to WGS in an Illumina novaseq 6000 system (Illumina, San Diego, United States) to obtain 150-bp paired-end reads. Raw reads were trimmed using Trimmomatic [35] and then assembled using SPAdes v3.12.0 [36]. MCR-CREC isolates were further sequenced by a Nanopore PromethION platform (Nanopore, Oxford, UK) following a 10-Kbp library protocol. The hybrid assembly of both short Illumina reads and long PromethION reads was performed using Unicycler v0.4.8 [37]. The assembled contigs were circularized by Circlator [38] and plasmid circularity was confirmed by PCR (Table 1) followed by Sanger sequencing. The assembled genomes were annotated using Prokka v1.17 [39] and were analyzed using Centre for Genomic Epidemiology (CGE, https://cge.cbs.dtu.dk/services/) for detecting the presence of antimicrobial resistance (AMR) genes (ResFinder v3.2) [40] and plasmid replicon typing (PlasmidFinder v2.1) [41]. The pair-wise average nucleotide identity (ANI) between the queries and type/protype genomes of *Enterobacter* species was determined by FastANI (https://github.com/ParBLiSS/FastANI), with a cut-off of 95% [42]. PubMLST (https://pubmlst.org/) was used for multilocus sequence typing (MLST) analysis, and ParSNP v1.2 [43] was used to align the core genomes. The synteny analysis was performed and visualized by using Easy Figure 2.2.2 [44]. Comparative analysis of plasmids was determined using the BLAST Ring Image Generator (BRIG) [45]. To evaluate the presence of a plasmid in draft genomes, contigs were blasted against the reference using Blastn v2.9.0 [46] to calculate the coverage.

**Table 1.** Primers used in this study.

Target	Primer name	Sequence (5’−3’)	Product size (bp)	Source
**Plasmid circularization**				
pECC27-MCR, pECC65-MCR	27-2-R	TTGACACCAGTTATCTTGATGTCGT	468	This study
	27-2-F	TAGAGTTCAAGTAAAGATCACGGCA		
pECC27-NDM, pECC65-NDM	27-3-R	CCAGACCCAATTACCAATAACTTC	455	This study
	27-3-F	CGCGGTAAAGCAATATACATCTTTA		
pECC30-MCR, pECC46-MCR, pECC47-MCR, pECC48-MCR, pECC116-MCR	30-2-R	CACCAGTTATCTTGATGTCG	460	This study
	30-2-F	TTCAAGTAAAGATCACGGCA		
pECC30-NDM	30-4-R	CCCGTTTGTACGAAGTCG	486	This study
	30-4-F	ACTTTTGACCATCGGCAC		
pECC45-MCR	45-2-R	AGGCTGATAATCTCACAGGA	453	This study
	45-2-F	CAGTATTGCGAGTTGAAGAG		
pECC45-NDM	45-3-R	TACTCTTTCCGGGAAGGG	466	This study
	45-3-F	TCATGGCAAACTGAAACG		
pECC45-IMP	45-4-R	TAACATCCTTGATAGCCTGGT	449	This study
	45-4-F	AAGTGTTATCTCTGGGAATCC		
pECC46-NDM, pECC116-NDM	46-3-R	GATTGCCATTCTGGAACTGAT	460	This study
	46-3-F	CACTATTTATATGCAGGCAGC		
pECC46-IMP, pECC47-IMP, pECC48-IMP	46-4-R	TAACATCCTTGATAGCCTGGT	449	This study
	46-4-F	AAGTGTTATCTCTGGGAATCC		
pECC47-NDM	47-3-R	CCTACTCTTTCCGGGAAG	472	This study
	47-3-F	GCGATCATGGCAAACTGA		
pECC48-NDM	48-3-R	AAGGCCGCCAGGTTAATC	468	This study
	48-3-F	CCCATGATGAAAAATGCC		
pECC60-NDM	60-5-R	GCATCCCTTTCCCTTTCAGCAAA	578	This study
	60-5-F	CACAACTGATTTCAATACCTGAGTGG		
pECC60-MCR	60-7-R	GCACGCAAGCTCAATGTACTGG	328	This study
	60-7-F	AGGCTCTTTAACGGCGAACGG		
pECC72-MCR	72-2-R	CACCAGGCGATTGTAGGCATGA	579	This study
	72-2-F	TTCAGGAGCAGGTAACGGGATTTC		
pECC72-NDM	72-4-R	TCGTTCTATTGGAATCACCGCTCT	380	This study
	72-4-F	AGCCATGCTGGTCTCCTTGTG		
pECC59-MCR	59-3-R	TCCATCTCCATTTCCCAG	474	This study
	59-3-F	ACGATAACCAACTGGCGA		
pECC59-NDM	59-4-R	AAGGGCGGTTGAACTTCC	472	This study
	59-4-F	AATGCGATCATGGCAAAC		
**Gene detection**				
*mcr-9.1*/*−9.2*	*mcr-9*-F	GCTTGTCGCCTTCCATATCATT	475	This study
	*mcr-9*-R	ATTATAGACGCTGGTGCTTACG		
*mcr-10*	*mcr-10*-F	CGGTTCGGTGGTGAGTTACG	1263	This study
	*mcr-10*-R	GCATTATGCTGCAGACACGC		
*bla*_IMP-4/IMP-26_	IMP-F	GCAGCAGAGCCTTTGCCAGATT	664	This study
	IMP-R	TTCCGCCCGTGCTGTCACTAT		
*bla*_NDM-1_	NDM-F	AGCTCGCACCGAATGTCTG	342	This study
	NDM-R	CATTGGCGGCGAAAGTCAG		

A total of 3,559 genomes of ECC were retrieved from the NCBI RefSeq database as of October 1 2021, and were subjected for the identification of *mcr-9/mcr-10* positive isolates using Abricate v1.0.1 (https://github.com/tseemann/abricate) and ResFinder v3.2 [40].

### Conjugation assay

In order to determine whether colistin and carbapenem resistance was transferable, conjugation assays were carried out in LB broth with using *E. coli* EC600 as the recipient according to a method previously described [16]. Briefly, logarithmic-phase cultures of donor and recipient cells were added to fresh LB broth and incubated overnight without shaking. Transconjugants were selected on LB agar plates containing 600 mg/L rifampicin plus 2 mg/L meropenem/imipenem and/or 2 mg/L colistin. The presence of carbapenemase and/or *mcr* genes in transconjugants was conﬁrmed using PCR and Sanger sequencing, and the species of transconjugants was confirmed using MALDI-TOF Mass Spectrometry (Hexin Instrument Co., Ltd, Guangzhou, China).

### Plasmid stability assay

Plasmid stability assay was performed as previously described with slight modifications [47]. The donor strains were grown in antibiotic-free LB broth. Cultures grew at 37°C in a shaking bath, followed by 1:1000 dilution in fresh antibiotic-free LB broth, and passaged for 10 successive days. Cultures of day 10 were serially diluted and plated on antibiotic-free LB agar plates, and incubated overnight. Fifty colonies were randomly picked from plates for each isolate. The presence of carbapenemase and *mcr* genes was validated by colony-PCR.

### Plasmid incompatibility assay

Plasmid incompatibility assay was performed as previously described [47]. Brieﬂy, the transconjugants bearing both carbapenemase-encoding and MCR-encoding plasmids were cultured in antibiotic-free LB broth and were grown at 37°C in a shaking bath. Overnight cultures were serially diluted and plated on antibiotic-free LB agar. Ninety-four colonies of each culture were selected for the detection of carbapenemase and *mcr* genes by PCR. Plasmids were considered incompatible when more than 80% of colonies lost either or both of carbapenemase-encoding and MCR-encoding plasmids.

### Accession numbers

The genome sequences have been deposited into GenBank. The detail accession numbers are listed in Table S1.

## Results

### Prevalence of MCR-ECC among ECC population

**Figure 1.**
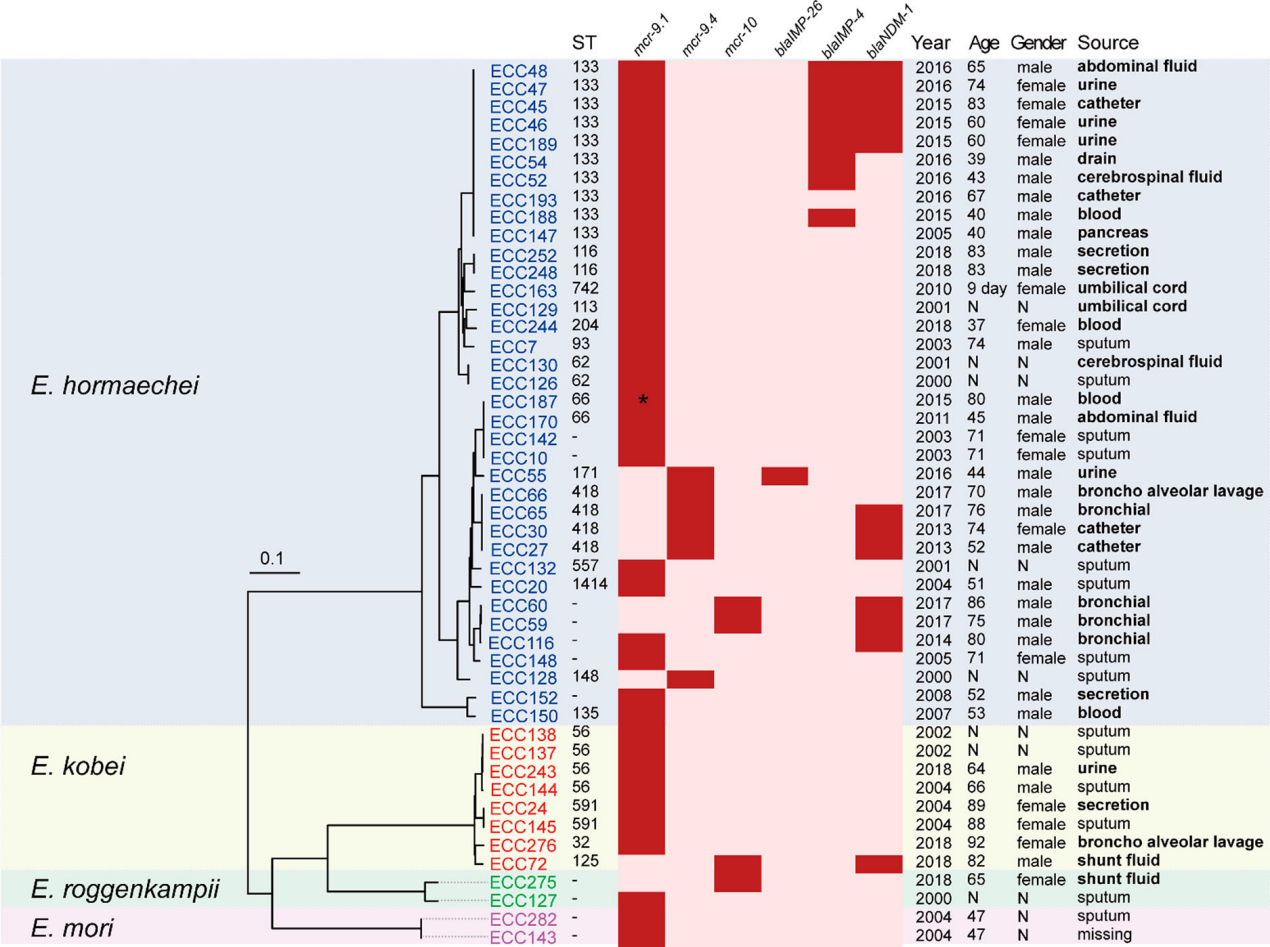
**Phylogenetic analysis and characteristics of the 48 MCR-ECC isolates collected in this study.** A core-genome phylogenetic tree including the 48 MCR-ECC isolates was constructed and mid-point rooted. The isolates clustered into four clades corresponding to four species (blue: *E. hormaechei*, yellow: *E. kobei*, green: *E. roggenkampii*, purple: *E. mori*). The ST of each isolate is shown, and the novel ST is indicated by a dash. The absence (in pink) or presence (in red) of colistin and carbapenem resistance genes is diaplayed by a heatmap, and a truncated mutant of *mcr-9* was marked with an asterisk. Clinical information of the isolates and patients are listed. The sterile-site specimens were in bold.

We collected 230 non-repetitive clinical ECC isolates at a tertiary hospital between 2000 and 2018. The isolates were previously assigned to eight species: *E. hormaechei* (*n* = 164), *Enterobacter kobei* (*n* = 26), *Enterobacter roggenkampii* (*n* = 17), *Enterobacter cloacae* (*n* = 13), *Enterobacter asburiae* (*n* = 2), *Enterobacter ludwigii* (*n* = 3), *Enterobacter bugandensis* (*n* = 2), *Enterobacter mori* (*n* = 2), and *Enterobacter cancerogenus* (*n* = 1) [34]. Most of the isolates were obtained from sputum (20.9%, 48/230), followed by urine (14.8%, 34/230), secretion (12.2%, 28/230), and blood (10.4%, 24/230) (Table S2). Forty-eight isolates were PCR positive for *mcr* genes (20.9%; 48/230) [48], of which 44 and four were positive for *mcr-9* (19.1%) and *mcr-10* (1.7%), respectively. The 48 MCR-ECC isolates belonged to four species: *E. hormaechei* (75.0%; 36/48), *E. kobei* (16.6%; 8/48), *E. roggenkampii* (4.2%; 2/48), and *E. mori* (4.2%; 2/48). They were recovered from various specimens according to the traceable metadata, and sputum accounted for the highest proportion (29.8%; 14/47), followed by urine (10.6%; 5/47). Of note, at least 28 isolates (59.6%; 28/47) were recovered from the sterile-site specimens (Figure 1) of patients. Of the patients positive for MCR-ECC with traceable metadata, 63.2% (24/38) were men, and 65.0% (26/40) were aged ≥ 60 years old (Figure 1). The first *mcr-9*-producing ECC isolate was detected in 2000, which is much earlier than the emergence of *mcr-10*-producing isolates (in 2017).

To understand whether *mcr-9*/*mcr-10* are prevalent in ECC population worldwide, we further identified the two genes in a collection of 3,559 ECC genomes retrieved from the NCBI RefSeq database. We detected 720 genomes positive to *mcr-9* and/or *mcr-10*, of which 657 (18.5%) and 70 (2.0%) were positive to *mcr-9* and *mcr-10*, respectively. Seven of the 720 genomes co-carried both genes. The prevalence ratio was comparable with that of our collection. The MCR-ECC isolates were assigned to 10 species: *E. hormaechei* (72.5%, 522/720), *E. roggenkampii* (7.5%, 54/720), *E. kobei* (6.7%, 48/720), *E. asburiae* (4.7%, 34/720), *E. cloacae* (4.2%, 30/720), *E. quasihormaechei* (3.2%, 23/720), *E. ludwigii* (0.6%, 4/720), *E. chengduensis* (0.3%, 2/720), *E. sichuanensis* (0.3%, 2/720) and *E. cancerogenus* (0.1%, 1/720) (Table S3). The results demonstrate that *mcr-9* and *mcr-10* were prevalent in ECC, where *E. hormaechei* was the predominant species.

### Emergence of MCR-CREC was later than that of MCR-ECC

The 48 MCR-ECC isolates harboured 7∼27 antibiotic resistance genes (ARGs) (median 23) (Table S1). Of these, 44 isolates carried *mcr-9.1* and its variants, including *mcr-9.1* (n = 37), *mcr-9.2* (n = 6), and an unknown variant showing 88.7% coverage and 100% nucleotide-acid identity in comparison to the sequence of *mcr-9.1* (Figure 1). Close inspection of the unknown variant identified an IS*15*-like insertion sequence, resulting in a 1437-bp fragment left, thus was designated as Δ*mcr-9.1* (Figure 1 and Table S1). The *mcr-10* gene was confirmed in four isolates (ECC59, ECC60, ECC72, and ECC275). Carbapenemase genes were detected in 16 isolates (13 *mcr-9*- and three *mcr-10*-positive isolates), including *bla*_NDM-1_ (n = 12), *bla*_IMP-4_ (n = 8), and *bla*_IMP-26_ (n = 1) (Figure 1 and Table S1). Five isolates (ECC45, ECC46, ECC47, ECC48, and ECC189) coproduced NDM-1 and IMP-4. The 16 MCR-CREC isolates were assigned to *E. hormaechei* (n = 15) and *E. kobei* (n = 1).

Among the 48 MCR-ECC isolates, 26 were resistant to colistin (54.2%), 15 to tigecycline (31.2%), and 16 to carbapenems (imipenem and/or meropenem). At least four isolates were resistant to two of the three classes (colistin + tigecycline: 4; colistin + carbapenems: 5; tigecycline + carbapenems: 8), and two were resistant to all. The MICs of the 48 MCR-ECC isolates are summarized in Table 2 and Table S1. Of note, all MCR-CREC isolates were detected in 2013∼2018, which is much later than the emergence of MCR-ECC (in 2000). We, therefore, suppose that MCR-CREC might be derived from MCR-ECC by capturing the carbapenemase genes in our collection.

**Table 2.** Susceptibility profiles and MICs for 48 MCR-ECC strains.

Antibiotics^a^	No. of resistant isolates	No. of intermediate isolates^b^	No. of susceptible isolates	MIC50 (mg/L)	MIC90 (mg/L)	MIC range (mg /L)
Ceftazidime	42 (87.5%)	1 (2.1%)	5 (10.4%)	> 64	> 64	< 0.0625 to > 64
Cefuroxime	39 (81.3%)	5 (10.4%)	4 (8.3%)	> 64	> 64	4 to > 64
Cefepime	23 (47.9%)	6 (12.5%)	19 (39.6%)	8	> 64	< 0.0625 to > 64
Imipenem	14 (29.2%)	1 (2.1%)	33 (68.8%)	0.25	4	0.125 to > 32
Meropenem	14 (29.2%)	0	34 (70.8%)	< 0.0625	16	< 0.0625 to > 32
Ciprofloxacin	40 (83.3%)	1 (2.1%)	7 (14.6%)	1	8	< 0.0625 to > 16
Ampicillin	48 (100%)	0	0	>128	>128	16 to > 128
Amikacin	6 (12.5%)	0	42 (87.5%)	8	> 128	0.25 to > 128
Gentamicin	33 (68.8%)	1 (2.1%)	14 (29.2%)	128	> 128	0.5 to > 128
Cefoperazone	20 (41.7%)	7 (14.6%)	21 (43.8%)	32	> 128	0.125 to > 128
Trimethoprim/Sulfamethoxazole	42 (87.5%)	0	6 (12.5%)	>320	>320	2.5 to > 320
Chloramphenicol	35 (72.9%)	3 (6.3%)	10 (20.8%)	> 128	> 128	8 to >128
Colistin	26 (54.2%)	0	22 (45.8%)	4	> 32	0.5 to >32
Tigecycline	15 (31.2%)	0	33 (68.8%)	0.5	4	0.25–8

**^a^**Breakpoints for antimicrobial resistance were determined according to CLSI guidelines, except that colistin and tigecycline were determined according to EUCAST guidelines.

^b^ MIC values not covered by the breakpoints are shown as intermediate here.

Among the 720 MCR-ECC genomes retrieved from GenBank, carbapenemase genes were detected in 388 genomes (53.9%), mainly including *bla*_IMP-4_ (19.8%; 77/388), *bla*_VIM-1_ (18.0%; 70/388), *bla*_VIM-4_ (14.2%; 55/388), *bla*_KPC-3_ (11.1%; 43/388) and *bla*_NDM-1_ (10.3%; 40/388) (Table S3). Most of the genomes were assigned to *E. hormaechei* (77.8%; 302/388), demonstrating that the convergence of carbapenemase and *mcr-9/10* genes was highly frequent in *E. hormaechei*.

### Prevalent clones shared by MCR-ECC and MCR-CREC

The population structure of the 48 MCR-ECC isolates was highly diverse, the top three STs were ST133, ST418, and ST56 (Figure 1) [48]. The 16 MCR-CREC were assigned to ST133 (n = 8), ST418 (n = 3), ST171 (n = 1), ST125 (n = 1) and novel STs (n = 3) (Figure 1).

A total of 152 STs were identified among the 720 MCR-ECC genomes retrieved from GenBank. ST133 and ST114 were the predominant clone (4.9% for each; 35/720), followed by ST171 (4.4%; 32/720) and ST93 (3.9%; 28/720) (Table S3). Sixty-two isolates were assigned to be novel STs. Ninety-eight STs were identified in the 388 MCR-CREC genomes, of which ST171 was the predominant clone (7.0%; 27/388), followed by ST93 (5.9%; 23/388), ST873 (5.4%; 21/388) and ST78 (4.9%; 19/388) (Table S3). Collectively, these data show that some prevalent clones were shared by MCR-ECC and MCR-CREC.

### An epidemic incHI2/2A plasmid highly contributes to the dissemination of mcr-9 in ECC worldwide

To identify how the carbapenemase and MCR genes were captured by MCR-CREC, we randomly chose 11 of 16 MCR-CREC isolates (eight positive to *mcr-9*, and three to *mcr-10*) for long-read sequencing to dissect the structure of plasmids. The sequencing results and assembly qualities are summarized in Table S4. The plasmidome of the 11 isolates can be classified into six groups according to Inc typing results, i.e. ECC59/ECC60 (IncHI2A, IncHI2, IncFIA, and IncX3), ECC27/ECC65 (IncFIB, IncHI2A, IncHI2, IncX3, IncFII, and IncFIB), ECC45/ECC46/ECC47/ECC48 (IncHI2, IncHI2A, IncX3, and IncN), ECC30 (IncHI2A, IncHI2, IncX3, and IncFIB), ECC72 (IncX3, IncFII, and IncFIB), and ECC116 (IncHI2A, IncHI2, IncFIB, IncR, and IncX3) (Table S4). The *mcr* and carbapenemase genes were located on different plasmids in each isolate, which were demonstrated by circularizing all plasmids carrying *mcr* and/or carbapenemase genes (see details in method) (Table S4).

**Figure 2.**
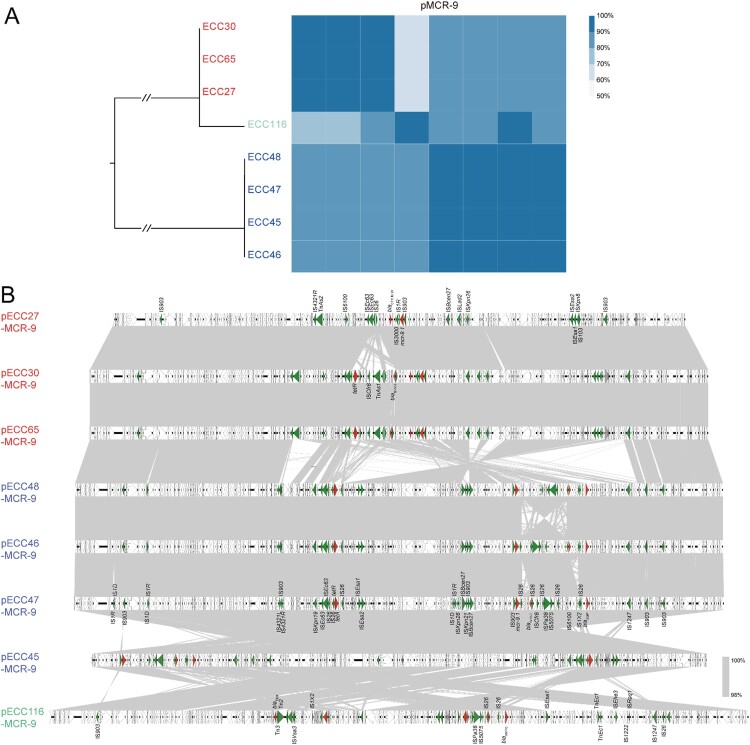
**Comparative genomic analysis on eight complete sequenced *mcr-9*-harboring MCR-CRE isolates.** (A) Core-genome phylogenetic tree accompanied by a similarity matrix of *mcr-9* plasmids. The isolates are coloured according to the STs (blue: ST133; red: ST418; green: a novel ST). The pairwise similarity between plasmids is shown by a heatmap, and is deﬁned as the coverage of homology regions for query plasmid (row-wise) and subject plasmid (column-wise). (B) Synteny analysis of eight circularized *mcr-9* plasmids. Identical regions (i.e. 100% similarity) are highlighted by grey rectangles. Arrows with direction indicate the sense of transcription of genes. Δ represents truncated genes.

The *mcr-9* gene detected on the eight long-read sequenced genomes (four ST133, three ST418, and one novel ST) located on IncHI2/2A plasmids with sizes ranging from 274,120 bp to 334,517 bp. Pairwise comparison of the eight circularized plasmids showed that the nucleotide similarity is accordant with the ST that plasmids carried by the same clone exhibited > 90% coverage and > 90% identity compared with each other, and pECC116-MCR-9 carried by the novel ST isolate shared < 70% coverage and < 90% coverage with the other plasmids (except for pECC45-MCR-9) (Figure 2A). Synteny analysis showed that the diverse regions among the eight plasmids were mainly caused by mobile genetic elements (Figure 2B), suggesting that they might have originated from a common ancestor with a conserved backbone, and had further co-evolved with the chromosome.

**Figure 3.**
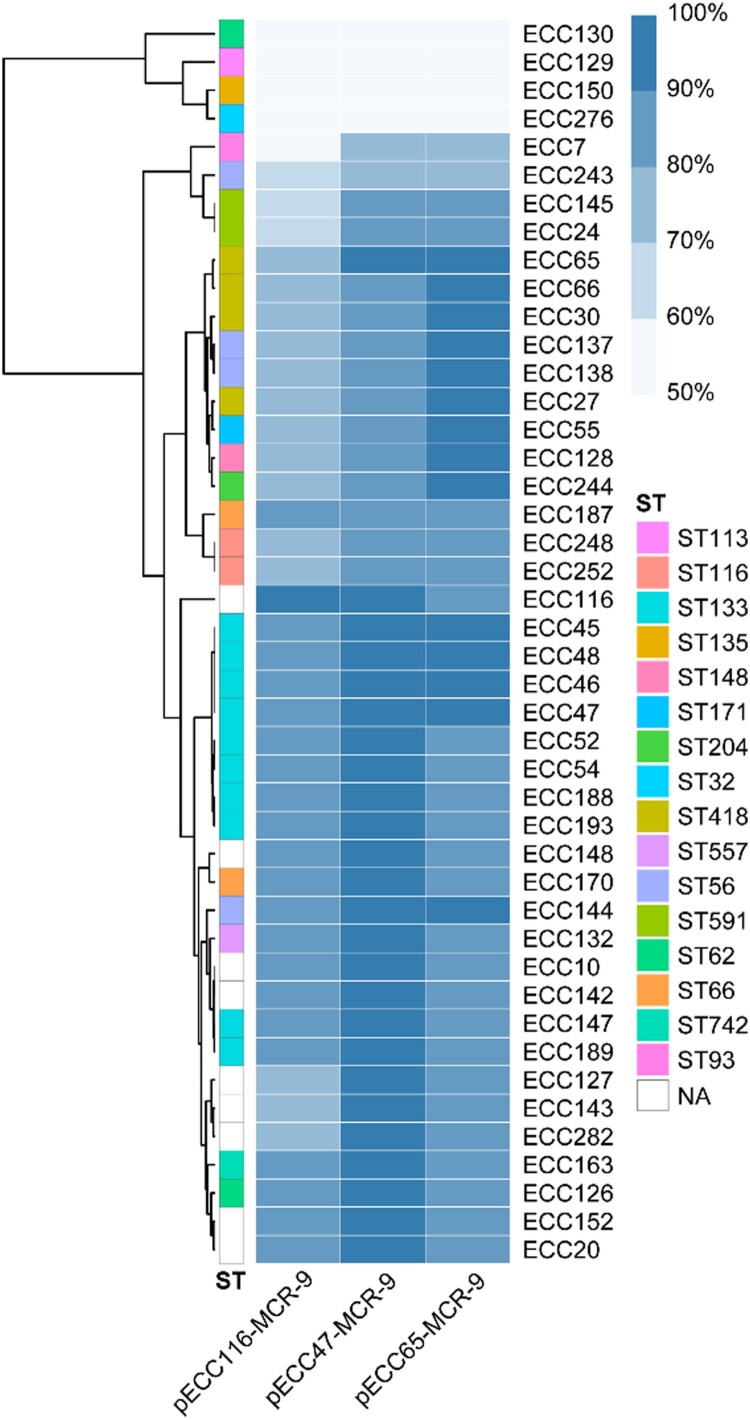
**Detection of pECC116-MCR-9, pECC47-MCR-9, and pECC65-MCR-9 in 44 *mcr-9*-carrying MCR-ECC.** The percentage length of virulence plasmid sequences are obtained by mapping short reads of the 44 isolates to the three *mcr-9* plasmids (pECC116-MCR-9, pECC47-MCR-9, and pECC65-MCR-9) used as references. The existence of plasmid is defined by that isolates having short reads mapped to ≥90% of the reference plasmid length. The isolates are clustered according to coverages using the Pearson method. STs of isolates are indicated, and NA represents the novel ST.

To further estimate the structure diversity of *mcr-9* plasmids in our collection, the 44 *mcr-9*-harboring draft genomes were mapped to three representative circularized plasmids, i.e. pECC47-MCR-9, pECC65-MCR-9, and pECC116-MCR-9. According to the mapping coverage, the *mcr-9* plasmids carried by the 44 isolates can be classified into at least six groups (Figure 3), and 33 isolates (75%) shared a high similarity (≥ 90%) with pECC47-MCR-9 and/or pECC65-MCR-9. We also identified 309 of the 657 *mcr-9*-positive genomes (47.0%) retrieved from GenBank showing ≥ 90% coverage to pECC47-MCR-9 and/or pECC65-MCR-9, and these genomes were obtained from 22 countries across six continents. The 309 genomes were assigned to seven species, of which *E. hormaechei* was the predominant one (n = 254) (Table S5). The results indicate that *mcr-9* dissemination among ECC was highly mediated by an IncHI2/2A epidemic plasmid.

### Highly conserved NDM-1- and IMP-4-carrying plasmids carried by MCR-CREC in China

**Figure 4.**
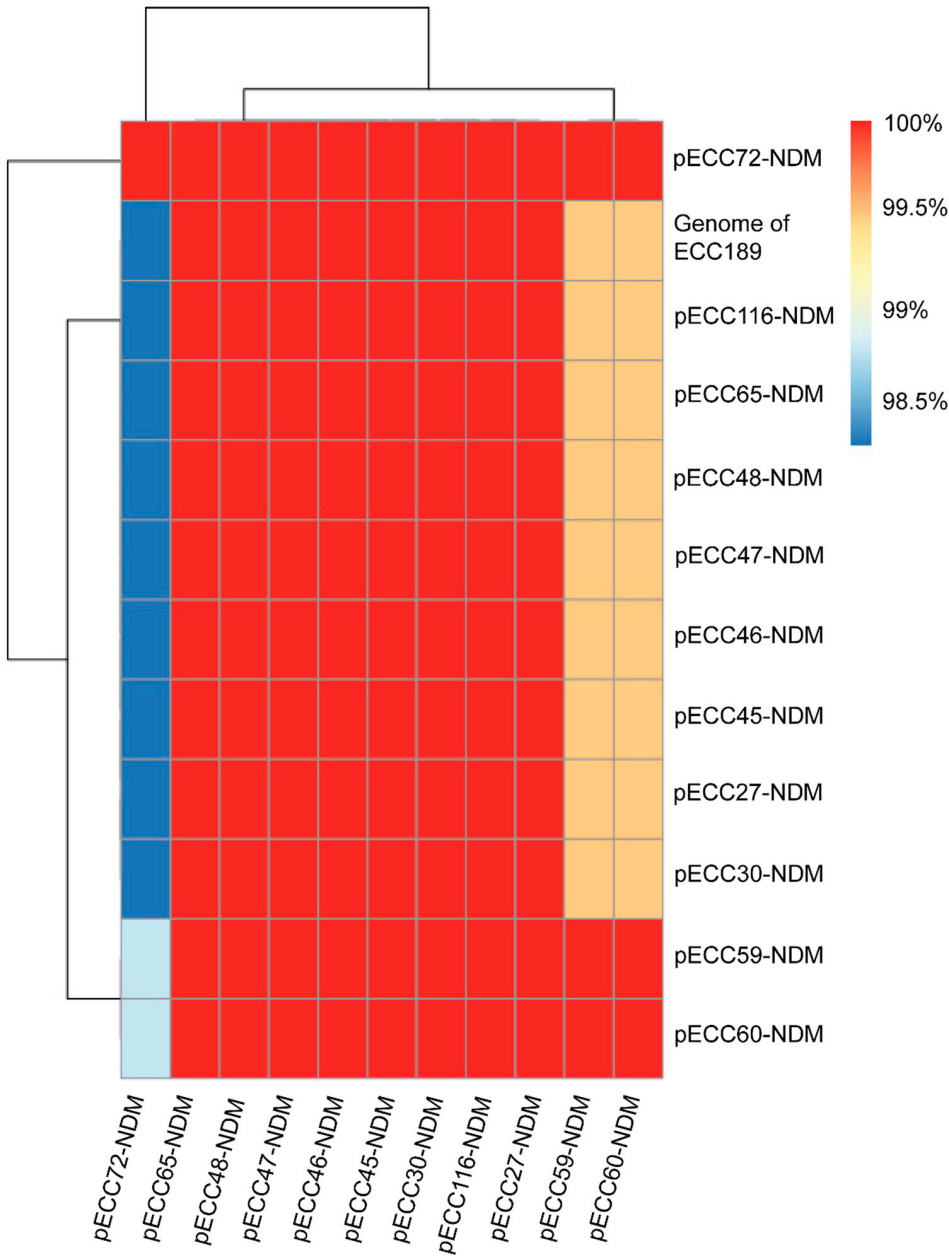
**Pairwise comparison of *bla*_NDM-1_-carrying plasmids.** Twelve *bla*_NDM-1_-carrying plasmids are included in the analysis, of which 11 are circularized, and the other one (Genome of ECC189) is analyzed using the draft genome. The similarity between sequences is deﬁned as the coverage of homology regions for query plasmid (row-wise) and subject sequences (column-wise).

All of the 11 hybrid assembled genomes carried a *bla*_NDM-1_ gene, and an IncX3-type NDM-1-carrying circularized plasmid with the size of about 53 kb was identified in each genome, with limited sequence variations (> 98% coverage and > 90% nucleotide identity) (Figure 4), suggesting the occurrence of cross-species horizontal transfer for the plasmid. The IncX3 plasmid additionally carried a bleomycin resistance gene *ble*_MBL_ and a beta-lactam resistance gene *bla*_SHV-12_. Blasting the representative pECC27-NDM against NCBI nt database identified 34 genomes sharing > 90% coverage and identity, and all of them were carried by *Enterobacterales*, of which 32 were isolated from China (Table S6), indicating that the IncX3-type NDM-1-carrying plasmid was an epidemic plasmid in *Enterobacterales* in China.

An IncN-type *bla*_IMP-4_-encoding circularized plasmid with the size of about 43 kb was detected in the four hybrid assembled genomes carrying a *bla*_IMP-4_ gene (ECC45, ECC46, ECC47, and ECC48) with highly limited sequence variations (100% coverage and > 99% nucleotide-acid identity). The four isolates belong to ST133, suggestive of a clonal dissemination. pECC45-IMP-4 was almost identical to pIMP-SZ1502 (GenBank accession no. KU051707) carried by an *E. coli* strain (99.9% identity and 100% coverage) (Figure S1). A quinolone resistance gene *qnrS1* co-located on the *bla*_IMP-4_-encoding plasmid. A pECC45-IMP-4-like plasmid was also detected in the remaining four IMP-4-positive genomes by mapping the reads to pECC45-IMP-4 (100% coverage and > 99% nucleotide-acid identity). These data indicate that the emergence of MCR-CREC was derived from MCR-ECC by capturing an IncX3-type NDM-1-carrying and/or an IncN-type IMP-4-encoding plasmid in China.

### The structure of mcr-10-carrying plasmids is highly diverse

A 64,293-bp IncFIA-type circularized plasmid was detected in two of the three hybrid assembled *mcr-10*-positive genomes (ECC59 and ECC60), since their plasmids (pECC59-MCR-10 and pECC60-MCR-10) were almost identical (100% coverage and 99.99% nucleotide-acid identity), leaving four core-genome SNPs. They were different from a previously reported 71,775-bp IncFIA-type plasmid pMCR10_090065 (GenBank accession no. CP045065) with 25% coverage and 99.92% identity (Figure S2) [14]. Blasting in the nt database showed that the IncFIA-type *mcr-10* plasmid shared the highest similarity (51% coverage and 98.2% identity) with a 160,042-bp IncFIA-FII-type plasmid pRHBSTW-00675_2 (GenBank accession no. CP056767) carried by an *E. cloacae* strain isolated from the wastewater. A 137,379-bp IncFIB-FII-type *mcr-10* circularized plasmid (pECC72-MCR-10) was identified in ECC72, and the most homologous plasmid identified in the nt database was a 240,613-bp IncFIB-FII-IncR-type plasmid pRHBSTW-00542_2 (GenBank accession no. CP056712) carried by an *E. asburiae* isolate, with 63% coverage and 99.44% identity.

The *mcr-10* of ECC275 was detected on a 35,290-bp contig encoding an IncFIB-type replicon. Mapping the sequencing reads of ECC275 to the three circularized plasmids (pECC59-MCR-10, pECC60-MCR-10, and pECC72-MCR-10) resulted in a low coverage ranging between 24.44% and 53.08%. When mapping the 70 *mcr-10*-positive genomes retrieved from GenBank to the three circularized plasmids, only four showed ≥ 90% coverage to pECC72-MCR-10 (Table S7). These results indicate that the structure of *mcr-10* plasmids might be highly diverse.

### Plasmids encoding mcr-9/mcr-10 are stable with NDM-1-encoding plasmids in ECC but incompatible in E. coli.

Conjugation assays were performed to evaluate the transferability of plasmids encoding MCR and carbapenemases. The *mcr* plasmids carried by most of MCR-ECC isolates were self-transmissible to *E. coli* EC600, including 32 *mcr-9* and four *mcr-10* isolates, and all plasmids encoding carbapenemase-encoding genes can be transferred successfully. The carbapenemase and *mcr* genes were able to be transferred simultaneously to EC600 from all MCR-CREC isolates.

The stability of plasmids encoding carbapenemases and MCR was evaluated in the 16 MCR-CREC by a 10-day passage. Fifty colonies of each isolate were tested (see details in method). All of the *mcr*-, *bla*_NDM-1_-, and *bla*_IMP-26_-harboring plasmids displayed high stability, as shown by the retention rates of over 80% at the end of the passages. While *bla*_IMP-4_-harboring plasmids showed low retention rates (10%∼74%) in six strains (ECC45, ECC47, ECC48, ECC52, ECC54, and ECC188), and ECC45 lost its IMP-4 activity after 10 days with 10% retention rate, suggestive of the instability for *bla*_IMP-4_-harboring plasmids in ECC (Table S8).

Plasmid incompatibility was further assessed in the transconjugants of the 16 MCR-CREC isolates. Ninety-four colonies of each isolate were tested (see details in method). The plasmids encoding *bla*_NDM-1_ or *bla*_IMP-26_ remained in all colonies overnight culture, while those with *mcr* or *bla*_IMP-4_ were completely lost (Table S9). These results suggest that the *mcr* and carbapenemase-producing plasmids were incompatible in *E. coli* EC600. The observed plasmid incompatibility is consistent with the current epidemiological data that in *E. coli mcr-9* and *mcr-10* was rarely detected and *bla*_NDM-1_ was prevalent.

### Genetic context of MCR and carbapenemase genes

**Figure 5.**
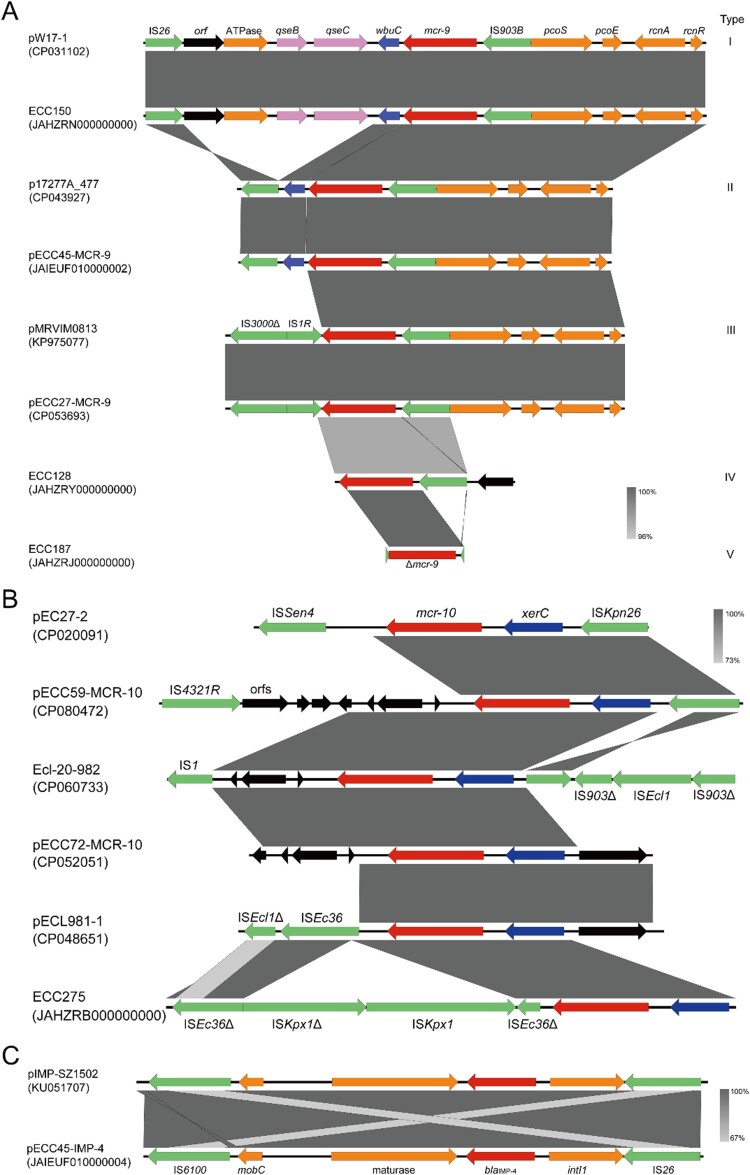
**Synteny analysis for genetic environments of (A) *mcr-9*, (B) *mcr-10*, and (C)** ***bla*_IMP-4_.** Identical regions are highlighted by dark grey rectangles (i.e. 100% similarity). Arrows with direction indicate the sense of transcription of genes, and Δ represents truncated sequences.

At least five types of *mcr-9* genetic contexts were identified in the 44 genomes (Figure 5A), and a module IS*903B* (intact or fragmented)*-mcr-9* was detected in all of them, implying the role of IS*903B* in the transmission of *mcr-9*. Type I genetic context was identical to that of pW17-1 (CP031102), and compared with the other types it exclusively encodes a two-component regulator *qseB/C* probably involved in *mcr-9* expression, an ATPase gene, and an *orf* with unknown function at downstream of *wbuC* (encoding a cupin fold metalloprotein). Type II might be derived from type I by the deletion of *orf-*ATPase-*qseB/C*, which is almost identical to that detected on p17277A_477 (CP043927). Compared with type II, IS*26*-*wbuC* was replaced by IS*3000*Δ-IS*1R* in type III as detected in pMRVIM0813 (KP97507). Different from the three types, *pcoS*-*pcoE*-*rcnA*-*rcnR* locating at the *mcr-9* upstream flanking was absent in type IV and V, and a gene encoding a hypothetical protein was detected in type IV instead. Note that, we were unable to determine the intact *mcr-9* genetic environments in 34 draft genomes due to the short *mcr-9*-bearing contigs available for the comparison.

The genetic context of *mcr-10* detected on pECC59-MCR-10 and pECC60- MCR-10 was identical (IS*kpn26-xerC*-*mcr-10-orfs-*IS*4321R*), while on pECC72-MCR-10 genes locating at upstream and downstream of *xerC*-*mcr-10* were replaced by those encoding hypothetical proteins without any insert sequences. An IS*Ec36* interrupted by the insertion of an IS*Kpx1* and a truncated IS*Kpx1* were located downstream of *mcr-10* in ECC275. The genetic contexts of *mcr-10* identified in this study are different from those described previously (Figure 5B) [16].

A highly conserved genetic environment of *bla*_NDM−1_ was found in all isolates that an IS*Aba125* interrupted by the insertion of an IS*5* lied upstream of *bla*_NDM-1_, and the fragment *ble*_MBL_-*trpF*-*dsbD*-*cutA*-*groS*-*groL*-*orf* lied downstream of *bla*_NDM-1_ (Figure S3). The *bla*_IMP-4_ was harboured by a class I integron in the four genomes with an identical structure (an IS*26*-truncated *intI*, *bla*_IMP-4_, a group II intron reverse transcriptase/maturase, a mobilization protein MobC, and an IS*6100*) (Figure 5C).

## Discussion

Carbapenems and colistin are the last resort antibiotics used for severe infections caused by multidrug-resistant bacteria. The worrisome fact is the emergence and spread of bacteria co-producing carbapenemases and *mcr* genes, largely limiting clinical treatment strategies and worsening outcomes. Understanding the occurrence and mechanism of such convergence of resistance to the last-resort antibiotics is imperative for making tailored strategies to control its further spread. In this study, we aimed to answer this question by systematically studying the co-existence of *mcr* and carbapenemase genes in ECC isolates collected between 2000 and 2018 in a tertiary hospital of China together with a global dataset retrieved from GenBank.

*mcr-9* has been detected in multiple species of *Enterobacterales* mainly isolated from clinical samples worldwide, e.g. *E. coli* [49], *S. enterica* [13], ECC [17], and *K. pneumoniae* [50]. In contrast, *mcr-10* was almost hosted in ECC and sporadically isolated from environment [16], animal [51] and clinical samples [52]. Previous studies have shown that *mcr-9* and *mcr-10* were frequently carried in ECC by using single centre or endemic data [14, 16, 22, 23]. To our knowledge, this is the first report demonstrating that *mcr-9*/*mcr-10* were prevalent in ECC globally with the ratio of ca. 20%, and *mcr-9* was much more prevalent than *mcr-10* (18.5% vs 2.0%). The *mcr-9* gene was firstly identified in a clinical *S. enterica* serotype Typhimurium strain isolated in 2010 [13], and *mcr-10* was in an *Enterobacter roggenkampii* clinical strain recovered in 2016 [14]. In this study, the earliest *mcr-9* and *mcr-10* isolates were obtained in 2000 and 2018, respectively, implying that *mcr-9* might have emerged much earlier than *mcr-10*. We further pinpoint that *E. hormaechei* was the major host of *mcr-9*. Accumulating epidemiological data suggest that *E. hormaechei* is the predominant species among ECC frequently obtaining multi-drug resistance and causing infections in the clinical setting [26, 53, 54]. Therefore, the high prevalence of *mcr-9* in *E. hormaechei* would further challenge the clinical treatment.

Different from the other *mcr* variants, the activities of *mcr-9* and *mcr-10* to colistin are much weaker in numerous isolates [13, 29, 55]. The first reported isolate carrying *mcr-9* or *mcr-10* was susceptible to colistin with an MIC of 0.25-0.5 mg/L, despite both genes did confer colistin MICs of > 2.5 mg/L when overexpressed in the laboratory strains [13, 14]. A recent study found that *mcr-9* expression was inducible in the presence of colistin when located upstream of the two-component system *qseB*/*qseC*, and the *mcr-9*-harboring isolates were susceptible to colistin when lacked the *qseB*/*qseC* regulatory operon [56]. In this study, *qseB*/*qseC* was detected at downstream of *mcr-9* in seven isolates resistant to colistin, further supporting the role of *qseB*/*qseC* in the induction of *mcr-9*-mdiated colistin resistance. While others lacking this two-component system at downstream of *mcr-9* also showed colistin resistance with even higher MIC values (≥ 32 mg/L), and no functional mutations (i.e. PhoP_D56_ and PhoQ_H277_) were detected in their chromosomes, suggestive of unknown mechanism involved.

Our analysis showed that the dissemination of *mcr-9* worldwide was mainly mediated by an IncHI2/2A epidemic plasmid. This is consistent with a previous study that IncHI2 was identified to be the dominant replicon type (90.1%; 64/71) of the *mcr-9*-carrying plasmids disseminating worldwide [57]. Notably, IncHI2/2A type plasmids are highly associated with multidrug resistance genes, which raising a concern that the *mcr-9*-carrying epidemic plasmid could become multidrug resistant in the future. In contrast*,* a highly diversity was found for the *mcr-10* plasmids that three different replicons (IncFIA, IncFIA-II, and IncFIB) were detected in the four *mcr-10*-carrying plasmids, and only one genome of the global dataset showed ≥ 90% coverage with one of the four plasmids. This is accordant with the finding of a recent study [33], and may be one of the causes for the less prevalence of *mcr-10* observed here.

Of greater concern, a high carbapenem resistance rate was detected in our MCR-ECC collection and also the global dataset, accounting for 33.3% (16/48) and 53.89% (388/720), respectively. These data demonstrate that the convergence of carbapenemase and *mcr-9/mcr10* genes was highly frequent in ECC. The discrepancy of plasmid stability observed between *bla*_IMP-4_-harboring and *bla*_IMP-26_-harboring plasmids might be caused by their different hosts that the host of *bla*_IMP-4_-harboring plasmids belongs to ST133, while that of *bla*_IMP-26_-harboring plasmid belongs to ST171. Our surveillance data showed that MCR-CREC emerged later than MCR-ECC in China, and the two populations shared prevalent clones (ST133 and ST418), we thus suspect that MCR-CREC might have been derived from MCR-ECC recently through obtaining plasmids encoding carbapenemases in clinical settings. This can be further supported by that the two prevalent carbapenemases detected in our collection, i.e. NDM-1 and IMP-4, were carried by an IncX3-type and an IncN-type epidemic plasmid with very limited sequence variations, respectively. Additionally, our results underpin that such convergence could be dependent on the host preference of plasmids, since *mcr-9/mcr-10*-encoding plasmids can be remained in ECC stably but not in *E. coli* though the plasmids encoding carbapenemase were stable in both.

Of clinical and epidemiological concern, various combinations of resistance to the last resort antibiotics were detected in MCR-ECC, and two of them were even resistant to colistin, carbapenems, and tigecycline. Such convergence would largely worsen clinical outcomes in the future, although the tigecycline resistance determinants could not be defined in this study. Additionally, a majority of MCR-CREC belonged to epidemic clones, e.g. ST171, ST93, ST133, and ST418 [48, 58, 59], and the plasmids encoding MCR or carbapenemases were able to remain stable in ECC and self-transmissible as shown here. Despite that it remains unclear whether the resistance convergence in these epidemic clones has established evolutionary advantages, continuous surveillance is imperative to prevent them from being high-risk clones.

In summary, our study revealed a high prevalence of *mcr-9* and carbapenemase genes co-existing in ECC, and the convergence was driven by epidemic plasmids. The data suggest that MCR-CRE could be derived from MCR-ECC, and multiple epidemic clones have mediated the dissemination of MCR-CRE worldwide, highlighting that effective measures should be taken to control its further spread.

## Supplementary Material

Supplemental Material
