# Supplementary material for: Frequent convergence of mcr-9 and carbapenemase genes in Enterobacter cloacae complex driven by epidemic plasmids and host incompatibility
Source: Emerg Microbes Infect. 2022 Aug 5;11(1):1959–72. doi: 10.1080/22221751.2022.2103456 (PMC9359198; doi:10.1080/22221751.2022.2103456)
Supplement: Supplemental Material [file TEMI_A_2103456_SM8821.zip › Supplemental data.docx]

**Supplementary Figure legends**

**Figure** **S1 Sequence comparison between different *bla*_IMP-4_-carrying plasmids.** The reference of each comparison is indicated in the core. Arrows indicate the sense of transcription of genes.

**Figure S2 Sequence comparison between different *mcr-10-*carrying plasmids.** The reference of each comparison is indicated in the core. Arrows indicate the sense of transcription of genes.

**Figure S3 Sequence comparison between different *bla*_NDM-1_-carrying plasmids.** The reference of each comparison is indicated in the core. Arrows indicate the sense of transcription of genes.
