# Supplementary figures and images for: Frequent convergence of mcr-9 and carbapenemase genes in Enterobacter cloacae complex driven by epidemic plasmids and host incompatibility
Source: Emerg Microbes Infect. 2022 Aug 5;11(1):1959–72. doi: 10.1080/22221751.2022.2103456 (PMC9359198; doi:10.1080/22221751.2022.2103456)

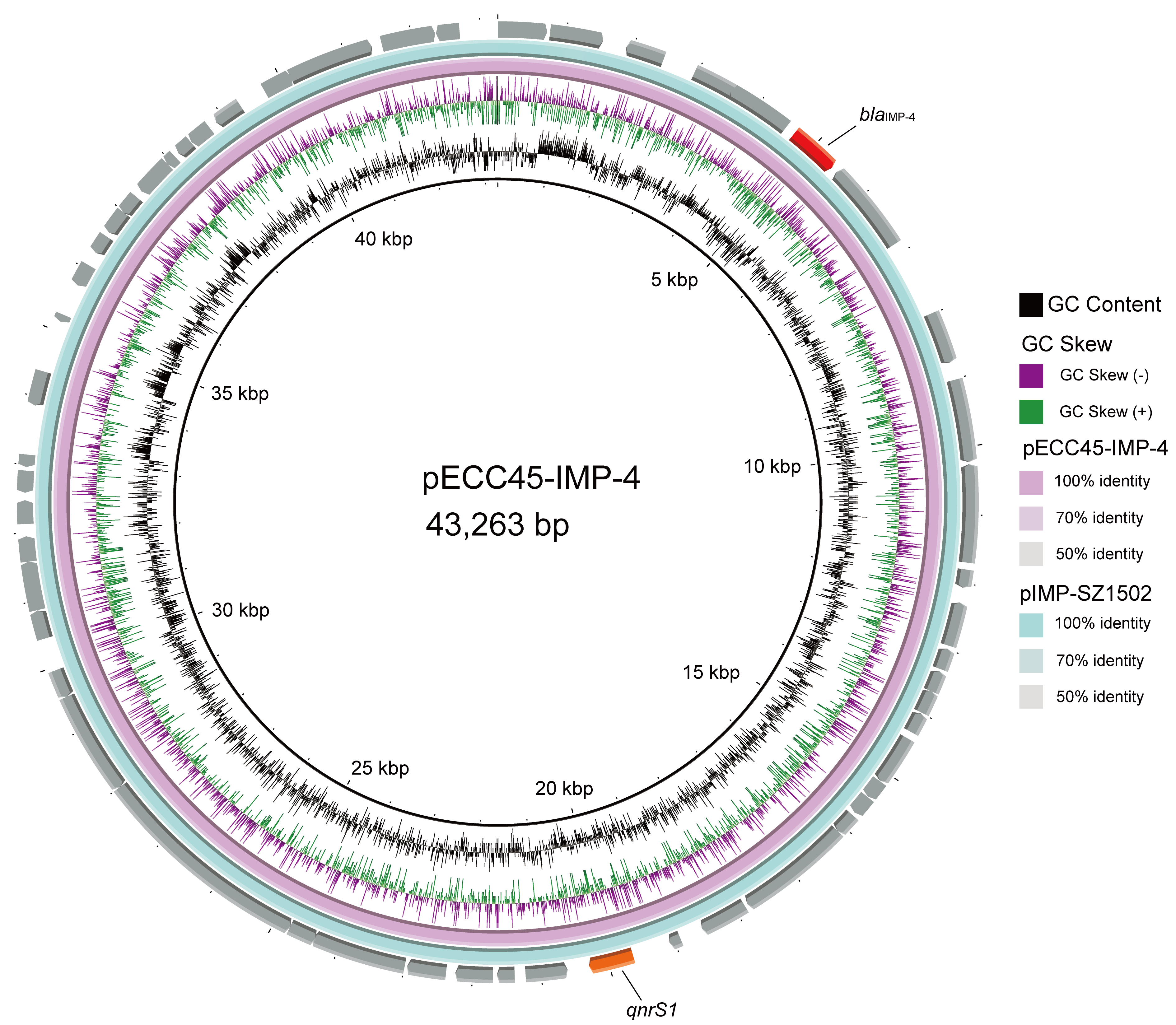

Supplement: Supplemental Material [file TEMI_A_2103456_SM8821.zip › Figure S1.tif]

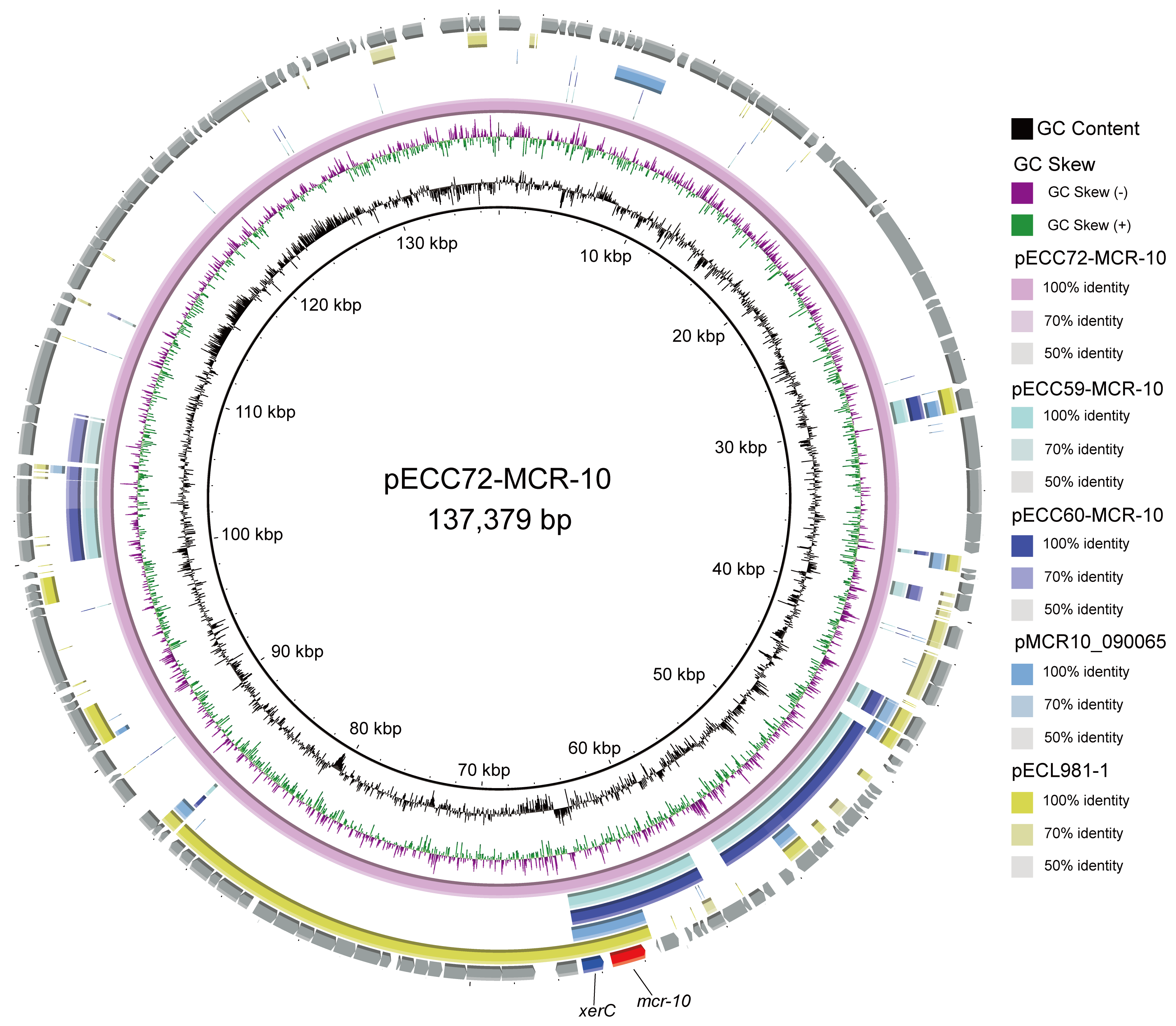

Supplement: Supplemental Material [file TEMI_A_2103456_SM8821.zip › Figure S2.tif]

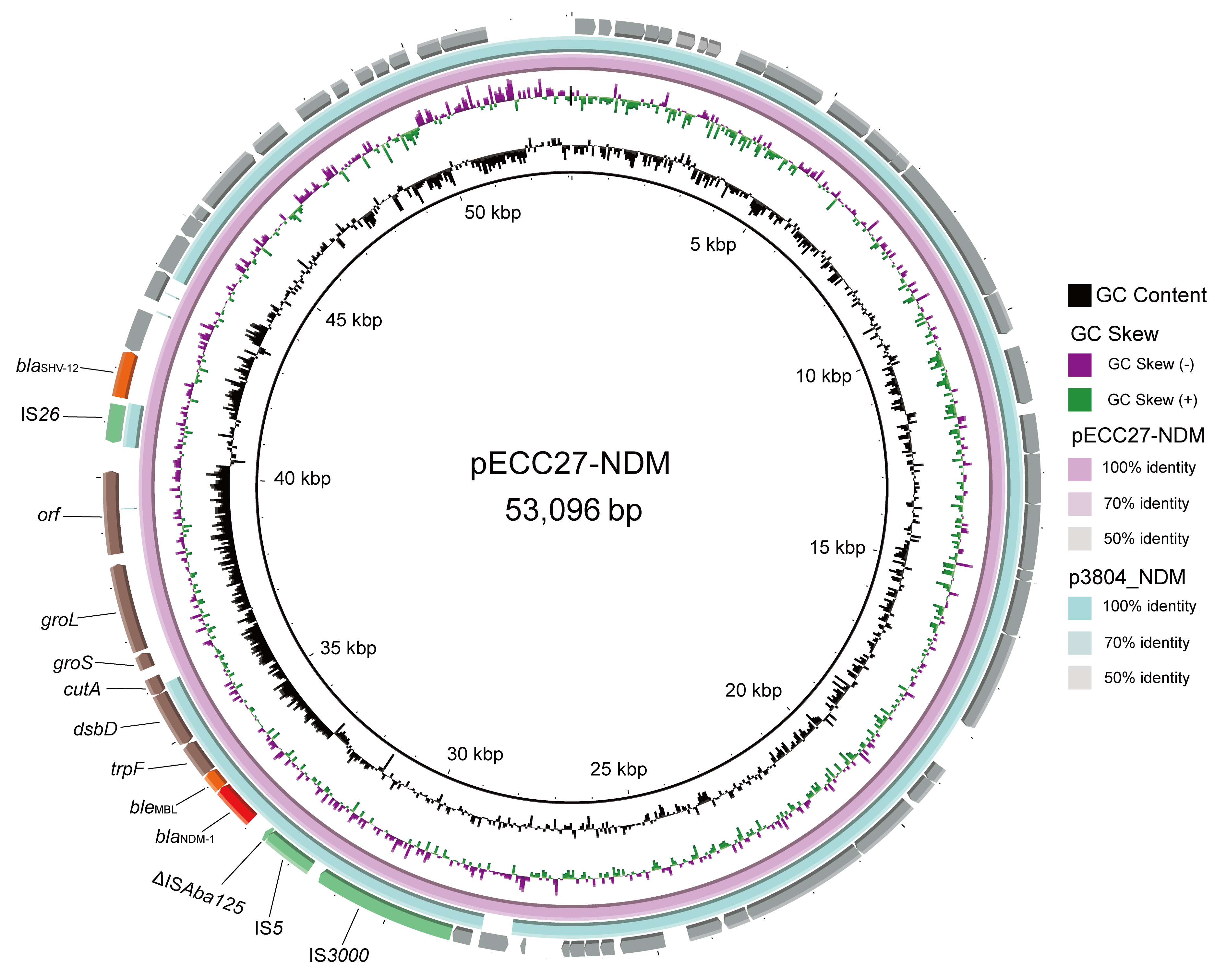

Supplement: Supplemental Material [file TEMI_A_2103456_SM8821.zip › Figure S3.tif]
